# Turner syndrome complicating osteogenesis imperfecta, thyroiditis and ankylosing spondylitis: a case report

**DOI:** 10.1186/s13256-026-06204-7

**Published:** 2026-07-30

**Authors:** Jiao Luo, Lili Jiang, Chunhua Shi, Lihua Duan

**Affiliations:** Department of Rheumatology and Clinical Immunology, Jiangxi Provincial People’s Hospital, The First Affiliated Hospital of Nanchang Medical College, Nanchang, 330000 People’s Republic of China

**Keywords:** Turner syndrome (TS), Osteogenesis imperfecta (OI), Ankylosing spondylitis (AS)

## Abstract

**Background:**

Turner syndrome (TS) is a chromosomal disorder characterized by monosomy of the X chromosome (45,X0) or structural aberrations in one of the X chromosomes in individuals with a female phenotype. Osteogenesis imperfecta (OI), commonly known as brittle bone disease, results from genetic abnormalities that lead to altered metabolism, reduced quantity, or structural defects in type I collagen, ultimately causing abnormal bone architecture, recurrent fractures, and progressive skeletal deformities.

**Case presentations:**

We report a 27-year-old Chinese woman with TS (45,X0) and a history of osteoporosis and uveitis who presented with 11 years of low back pain and one month of bilateral ankle swelling. She had previously undergone spinal fixation and bilateral hip replacement. Examination revealed blue sclerae, height 132 cm, and weight 26 kg. Laboratory findings showed elevated erythrocyte sedimentation rate (ESR, 52 mm/hour) and C-reactive protein (CRP, 75.9 mg/L), positive HLA-B27, and elevated thyroid antibodies. Imaging revealed sacroiliitis on computed tomography and enthesitis on ankle magnetic resonance imaging (MRI). Genetic testing identified a heterozygous collagen type I α 1 chain (COL1A1) mutation and an Xp22.33-q28 deletion. She met the modified New York criteria for ankylosing spondylitis (AS). Treatment with adalimumab led to clinical improvement with decreased erythrocyte sedimentation rate (ESR) and C-reactive protein (CRP) after one month.

**Conclusions:**

This case highlights the rare co-occurrence of TS, OI, and AS, emphasizing the importance of genetic and rheumatologic evaluation in complex presentations. We recommend annual clinical assessment for inflammatory back pain and, when clinically indicated, sacroiliac imaging (such as MRI or X-ray) to facilitate early diagnosis and management of concurrent rheumatologic conditions.

## Introduction

Turner syndrome (TS) is a chromosomal disorder characterized by the absence of one X chromosome (45 X0) or a structural abnormality in one of the chromosomes in phenotypic females. Individuals with TS exhibit a variety of clinical manifestations, such as short stature, ovarian dysgenesis, delayed puberty, infertility, congenital malformations, endocrine disorders (including autoimmune conditions and type 2 diabetes), and neurocognitive deficits. These individuals also experience heightened morbidity and mortality rates compared to the general population, with a delayed average age at diagnosis [1]. Patients with Turner syndrome are at risk for other genetic diseases; for example, they are predisposed to skeletal abnormalities such as Madelung deformity and scoliosis, which are linked to SHOX gene haploinsufficiency [1]. In individuals diagnosed with TS, there is a higher prevalence of acquired endocrine, autoimmune, and immunological disorders than what is typically observed in the general population [2, 3]. Beyond the well-documented risk of autoimmune thyroiditis and inflammatory bowel disease, Turner syndrome is also associated with a spectrum of other inflammatory disorders, including celiac disease, juvenile idiopathic arthritis, and less commonly, spondyloarthritis [4–7]. TS with ankylosing spondylitis (AS) is rare.

Osteogenesis imperfecta (OI), also known as brittle bone disease, is an autosomal or recessive connective tissue disease characterized by weak and malformed bones, blue sclera, dental dysplasia, and short stature. Type I collagen gene variant is the main cause of all types of OI. The X-ray manifestations of OI mainly include osteoporosis, multiple fractures and multiple bone malformations. OI and TS are both rare diseases, and the patient combined with two diseases is even rarer.

Here, this case report firstly describes a rare occurrence of OI and two autoimmune disorders (AS and autoimmune thyroiditis) in a patient with TS. The findings suggest that TS patients may be at risk for developing other genetic disease and multiple autoimmune diseases, such as AS.

## Case report

The patient was a 27-year-old Chinese woman with a past medical history significant for Turner syndrome (45 X0 karyotype at the age of 13) and osteoporosis, ocular uveitis, and spinal fracture who presented to the rheumatology clinic with the complaints of low back pain for 11 years and swollen ankle pain for 1 month. In 2013, the patient began to suffer from low back pain without obvious causes, and later developed hip pain and limited movement after falling. Relevant examinations were completed in the local hospital, and the patient was diagnosed as avascular necrosis of the femoral head and spinal fracture, and underwent spinal internal fixation and double hip femoral head replacement surgery. After discharge, the patient still had long-term pain after taking long-term calcium supplements such as alendronate and calcium tablets. The patient developed pain and swelling of both ankles for 1 month ago. The patient was admitted to our hospital for treatment on March to 12, 2024.

The patient’s temperature is 36.5°, pulse is 83 bpm, blood pressure is 129/78 mmHg, height was 132 cm and weight was 26 kg. The sclera was blue. Musculoskeletal examination was limited due to bilateral hip replacement; however, tenderness was elicited over the bilateral sacroiliac joints. Ankle examination revealed swelling, warmth, and tenderness over the bilateral tibiotalar joints. No peripheral joint deformities or enthesitis at other sites were noted. Neurological examination was unremarkable. Laboratory studies showed that erythrocyte sedimentation rate (ESR) was 52 mm/hour (normal range 0–20), C-reactive protein (CRP) was 75.9 mg/L (normal range 0.000–0.800) and hemoglobin was 98 g/L (normal range115–150). Assays for ANA, anti-dsDNA, rheumatoid factor were negative. Hepatitis B surface antigen was positive and Hepatitis B e antigen was negative. Hepatitis B DNA was negative. Routine serum chemistry tests and urinalysis were normal. Blood, urine and sputum cultures were negative. Serum protein electrophoresis was normal. serum estradiol, follicle stimulating hormone, growth hormone and luteinizing hormone levels were normal. Her thyroid function tests revealed that she had normal levels of free T3, T4 and of the thyroid stimulating hormone levels. Also, her anti-thyroglobulin antibody 471.50 IU/ml (normal range  0.00–115), anti-microsome antibody 485 IU/ml (normal range 0.00–32.0) and anti-thyroid peroxidase antibody 70.64 IU/ml (normal range 0–35). Her human leukocyte antigen typing was positive for HLA- B 27.

**Fig. 1:**
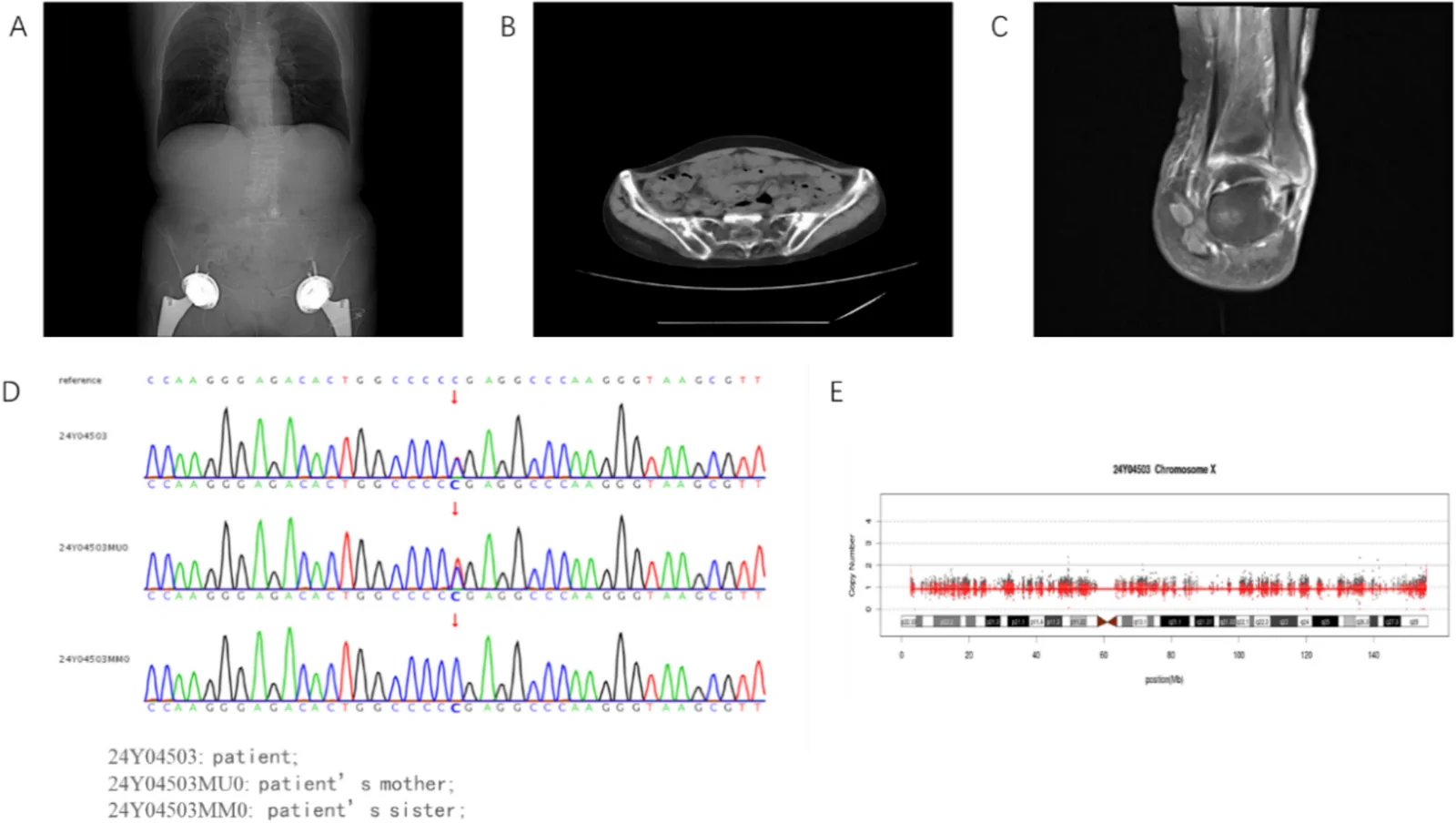
**A** Spinal X-ray: double hip replacement and compression fracture of the spine. **B** Sacroiliac CT showed changes in sacroiliac arthritis. **C** Magnetic resonance imaging of ankle joint revealed bone marrow edema of left tibia, distal fibula, talar, calcaneus and cuneiform bone, injury of anterior talofibular ligament and posterior talofibular ligament. **D** The variants of collagen type I α 1 chain (COL1A1). **E** Missing chromosome X

Pelvic ultrasonography demonstrated a rudimentary uterus. Radiological investigation showed severe compression fracture of the thoracolumbar and after double hip arthroplasty (Fig. 1A). Since the patient was unable to complete the sacroiliac magnetic resonance position, the patient's sacroiliac computed tomography (CT) showed changes in sacroiliac arthritis (Fig. 1B). MRI of the ankle joint revealed bone marrow edema of left tibia, distal fibula, talar, calcaneus and cuneiform bone, injury of anterior talofibular ligament and posterior talofibular ligament (Fig. 1C). Patient's genetic test showed collagen type I α 1 chain (COL1A1): NM 000088.4:exon4:c.358C > T:p.R120* variation. The mutant gene in the patient and her mother is heterozygous, while the sister's is homozygous (Fig. 1D). In addition, the 152.83 Mb region is missing at Xp22.33-q28 on chromosome X (Fig. 1E).

Overall, the diagnostic approach was guided by the patient's chronic inflammatory back pain (duration > 3 months, morning stiffness > 30 minutes, improvement with activity), imaging findings (sacroiliitis on CT), and positive HLA-B27. These findings fulfilled the modified New York criteria for ankylosing spondylitis [8]. The concomitant diagnosis of osteogenesis imperfecta was confirmed by genetic testing, which identified a heterozygous *COL1A1* mutation (c.358C > T, p.R120*). This mutation, along with the patient's blue sclerae, history of low-trauma fractures, and short stature, supported the diagnosis of OI. The presence of Turner syndrome was confirmed by the Xp22.33-q28 deletion identified on genetic testing. The patient was treated with adalimumab every two weeks and zoledronate, calcitol and calcium tablets for osteoporosis. Entecavir is used for anti-hepatitis B virus therapy. ESR was reduced to 21 mm/hour and CRP was reduced to 5.67 mg/L after 1 month of adalimumab treatment. We have summarized the timeline for this case in Table 1.

**Table 1:** Clinical timeline of the patient

Time	Event	Diagnosis/intervention
2013	Low back pain onset; fall with hip pain	Symptoms began
2013	Evaluation at local hospital	Diagnosed: avascular necrosis, spinal fracture
2013	Spinal fixation, bilateral hip replacement	Surgical intervention
Age 13	Short stature, delayed development	Diagnosed: Turner syndrome (45,X0)
2013–2024	Persistent pain	Long-term alendronate, calcium
March 2024	Admitted with back pain (11 years) + ankle swelling (1 month)	Hospitalization
March 2024	HLA-B27 + , sacroiliitis on CT, *COL1A1* mutation, Xp22.33-q28 deletion	Diagnosed: AS, OI
March 2024	Started adalimumab, zoledronate, entecavir	Treatment initiated
April 2024 (1 month)	Follow-up	ESR 21 mm/hour, CRP 5.67 mg

CT: Computed tomography. HLA-B27: Human leukocyte antigen B27. COL1A1: Collagen type I α1 chain. AS: Ankylosing spondylitis. OI: Osteogenesis imperfecta. ESR: Erythrocyte sedimentation rate. CRP:C-reactive protein

## Discussion

We present a unique case of a patient with TS who concomitantly exhibited OI, autoimmune thyroiditis and AS. The correlation between autoimmune disorders and chromosomal abnormalities, including TS, has been previously documented in the literature. However, the precise role of chromosomal abnormalities in the pathogenesis of autoimmune disorders remains to be elucidated. Thyroid abnormalities are commonly observed in individuals with TS [9, 10]. Radetti and colleagues demonstrated that 22% of the TS patients in their study (106 out of 478 patients) exhibited autoimmune thyroiditis. However, they did not observe any correlation between karyotype and the prevalence of thyroid autoimmunity in TS [9].

OI is also known as brittle bone disease. It is primarily inherited in an autosomal dominant manner, although a minority of cases exhibit autosomal recessive inheritance and sporadic “Variants”. Its pathogenesis is that gene variant leads to abnormal metabolism, decrease in quantity or structural abnormality of type I collagen, which leads to abnormal bone structure and eventually leads to repeated fractures and progressive bone malformations. At present, 21 pathogenic genes of OI have been reported. Among them, COL1A1 or collagen type I α 2 chain (COL1A2) variant is the main cause of OI. OI was divided into seven types according to clinical characteristics [11, 12]. Type 1 of OI is characterized by blue sclera, dentine hypoplasia, progressive hearing loss, skin tendon and ligament relaxation. Only type 1 is common in adults. Given the early-onset spinal rigidity, recurrent low-trauma fractures, and blue sclerae noted on exam, clinical suspicion for osteogenesis imperfecta (OI) arose. While OI is typically associated with COL1A1 or COL1A2 mutations, these genes are critical for type I collagen structure. Pathogenic variants can lead not only to bone fragility but also to alterations in the extracellular matrix of entheseal sites, potentially influencing the phenotype of inflammatory arthritis. This patient’s genetic test showed COL1A1 variant and the variant site is on chromosome 17. From the clinical manifestations, it is classified as type 1. McKiernan reported the results of case study of 111 patients with type I of OI, showing pain, stiffness, and instability in the weight-bearing joints of the lower extremities as the main clinical manifestations. Nearly half of the respondents (44%) had a clinical diagnosis of "arthritis". Among them, OA was 67%, "unknown cause" arthritis was 37%, fibromyalgia was 6% and inflammatory joint disease (RA, PSA) was 4%. These patients present with multiple fractures including calf, forearm, spine, ankle, hip, and so on [13]. The patient's mother is heterozygous of COL1A1. His sister is homozygous of COL1A1. The patient's CLOIAI variant came from her mother. His mother also had blue sclerae. We considered whether patients who acquired the COL1A1 variant during the genetic process were more likely to develop TS. This is worthy of further study. Interestingly, the mother did not have significant pathological fractures or osteoporosis. TS with OI may increase the risk of osteoporosis and fracture. In addition, the patient reported in this case had low back pain, and sacroiliac CT showed bone destruction, hyperplasia and sclerosis of the sacroiliac joint. HLA-B27 was positive, and erythrocyte sedimentation rate (ESR) and C-reactive protein were elevated. It meets the diagnostic criteria for AS. AS is a chronic, inflammatory spondyloarthritis (SPA). distinguished by inflammatory back pain resulting from sacroiliitis and spondylitis, often accompanied by peripheral arthritis, enthesitis, and acute anterior uveitis [14]. The HLA-B27 molecule is strongly associated with the SPA. HLA B27-related diseases such as anterior uveitis, psoriatic arthritis (PSA), and juvenile rheumatoid arthritis (JRA) have been documented to exhibit an association with TS [5, 15]. In this case, Patients with OI who are human leukocyte antigen (HLA)-B27 positive have more severe osteoporosis than those who are negative [16]. Although HLA-27-associated AS combined with TS and HLA-27-associated AS combined with OI have been reported, this case is the first to be combined with all three diseases. This case is a unique contribution to understanding the potential overlap of heritable disease and inflammatory spondyloarthritis. Whether these three diseases are genetically related still needs to be further studied. In addition, this case indicates that physicians should consider the potential presence of AS in patients presenting with low back pain or joint pain, particularly those with TS or OI.

## Conclusion

This case highlights the diagnostic complexity when Turner syndrome, a genetic condition with inherent autoimmune predisposition, co-occurs with osteogenesis imperfecta, a disorder of collagen structure, in a patient presenting with ankylosing spondylitis. The association of COL1A1 mutations with both bone fragility and potential enthesopathy suggests a possible interplay between genetic matrix defects and inflammatory pathways. This case emphasizes the importance of a comprehensive genetic and rheumatologic evaluation in patients with atypical or severe presentations of spondyloarthritis, particularly in the context of underlying genetic syndromes.

## Data Availability

The data that support the findings of this study are available from the corresponding author upon reasonable request.
